# A heutagogical approach for the assessment of Internet Communication Technology (ICT) assignments in higher education

**DOI:** 10.1186/s41239-021-00290-x

**Published:** 2021-10-18

**Authors:** Michael Lynch, Todd Sage, Laurel Iverson Hitchcock, Melanie Sage

**Affiliations:** 1 University at Buffalo School of Social Work, 685 Baldy Hall, Buffalo, NY 14260 USA; 2 Department of Social Work, University of Alabama at Birmingham, 3154 University Hall, 1402 10th Avenue South, Birmingham, AL 35294-1241 USA

**Keywords:** Heutagogy, Internet Communication Technologies (ICT), Authentic audiences, Self-directed learning, Assessment, Evaluation

## Abstract

Pedagogical foundations exist for incorporating technology in instruction; however, these foundations have not kept pace with technology's evolution. Through the use of Information Communication Technologies (ICTs), students now can share content directed at external audiences, i.e., audiences other than the instructor. These audiences are referred to as authentic audiences as they are public-facing and exist outside of the classroom. The existing literature offers evidence of student satisfaction with assignments directed at appealing to external audiences, however, the literature provides no comprehensive pedagogical rationale for assignments directed at authentic audiences wherein the goals are self-determined. The authors discuss the theory of heutagogy, the study of self-determined learning, as an approach for assessing assignments that utilize ICTs and are directed at authentic audiences. Finally, the authors offer an approach for the assessment of these assignments, including a rubric.

## Introduction to problem

College instructors increasingly offer assignments that harness the internet for communication or dissemination, such as through infographics, podcasts, the creation of blogs, or social networking. Reasons broadly include enhancing student engagement, connecting students with public audiences, and increasing digital literacy. Existing literature offers evidence of student satisfaction with assignments in which they create material for public audiences using Internet Communication Technologies (ICTs) (Armstrong et al., 2009; Hitchcock & Battista, 2013; Wopereis et al., 2010), as well as the benefits of authentic assessment in which participants are evaluated in instructor-designed real-world activities that mirror future vocational work (Gulikers et al., 2004). However, a literature gap exists relative to pedagogical reasons for asking students to publicly share their work using ICTs, and around the best practices for designing and assessing ICT assignments. Technology is increasingly central to higher education, and even more so in light of the COVID-19 pandemic, where many classes have moved entirely online, requiring instructors to use technology in increasingly novel ways (Johnson et al., 2020). Theoretical lenses such as heutagogy offer an opportunity to consider the implications of these types of assignments in light of learning outcomes and assessment. Briefly, heutagogy is an instructional approach focused on learner self-determination.

One hesitation when using ICTs in the classroom is about how to assess and assign a grade to this kind of work, especially when students are designing content for a specific audience besides the teacher. We attempt to answer this dilemma by presenting a pedagogical guide for educators seeking to incorporate ICTs in higher education curricula using a heutagogical lens. First, we describe the importance of ICTs in the 21st Century classroom, particularly in light of the impact of the COVID-19 pandemic. Next, we describe heutagogy, which provides instructors in higher education with a rationale for utilizing ICTs for assignments that support student engagement with authentic audiences. Finally, we present an assessment approach for public-facing ICT assignments. This discussion fills a gap in knowledge and practice related to how college educators assess ICT assignments in higher education (Sosa & Manzuoli, 2019).

### ICTs in the 21st century classroom

Broadly defined, ICTs are technologies that allow for information sharing and communication with others over the internet (Sosa & Manzuoli, 2019). Rather than just offering a new tool, ICTs structurally change the way that information is accessed, diffused, and adopted (Reid, 2002). Learners are increasingly influenced by ICTs outside the classroom, and are often already self-directed in the ways that they navigate these tools to learn about issues of interest to them (Sutherland, 2004). Just as with prior innovations that shift knowledge dissemination, such as the printing press and telephone, ICTs have brought forth conflicting positions about whether these tools are helpful in the classroom (Reid, 2002). Some of these concerns stem from the role of the teacher when these technologies are used (Reid, 2002; Sutherland, 2004), which this paper helps to address.

Examples of ICTs in the classroom include the use of social networking sites (i.e. Twitter and Instagram) and websites that can host digital content as well as audio and video recording software for creating digital content. In higher education, ICTs are often used to enable learners to develop products designed for public consumption, like podcasts, websites, blogs, or social media comments in spaces like Twitter, or bookmark material for sharing in places like Pinterest. ICTs can be integrated into assignments as an add-on such as asking students to post comments about a research paper on Twitter, or they can be Learner-Generated Digital Media (LGDM) assignments which require the student to conceptualize and create digital media content for an assignment such as a podcast, infographic or video (Reyna et al., 2017).

The latter, LDGM assignments allow students to create products for an authentic, public audience, rather than the artificial instructor-only audience. Authentic audiences are interested in the subject matter produced by the student, and authentic audiences are shown to enhance student engagement and performance (Herrington et al., 2014; Newmann, 1995; Newmann et al., 1996). Using ICTs with authentic audiences encourages students to consider their professional voice while promoting technological proficiency. It offers opportunities for creative thinking beyond typical specific assignment structures often used in classroom papers. Considerations related to the professional and ethical public presentation of self are essential for public servants, such as teachers, social workers, and health professionals; these assignments offer hands-on reflective practice.

Prior research on authentic audiences focuses on how this approach meets the goals of student-centered or community-based learning (Newmann et al., 1996). ICTs offer additional opportunities to reach very particular audiences. For example, students can create content directed at specific professional or regional groups or hard-to-reach populations by utilizing social media groups or hashtags. The flexibility offered to students makes assessment difficult because the student may have more content and context expertise about the target population than the instructor, and the products may vary widely based on the project (Cochrane & Antonczak, 2013). Additionally, technical rubrics, such as those that assess citation style or the inclusion of specific content, are not easily transferable to the range of ICT assignments that can be used in the classroom.

Not only do ICTs facilitate sharing with authentic audiences, but they also allow for direct communication and collaboration between students and their audiences through digital tools such as private messaging and commenting features. Thus, students can actively engage with their audience across an assignment's development, rather than passively sharing, which transforms the audience into supporters or mentors (Ito et al., 2013). Rather than very clear instructions afforded by typical college assignments, these assignments require critical thinking and the ability to manage ambiguity. These authentic connections offer learners access to diverse and multidisciplinary ways of thinking and feedback, collaboration, self-reflection, along with meaningful associations and networking with professionals or other types of interested community beyond the typical classroom, which often focuses more on direct knowledge transfer.

The importance of ICTs is more apparent given the changing landscape of higher education, which faces contemporary pressures related to the COVID-19 pandemic and social justice movements, where content does not yet exist in textbooks. Students are very exposed to public dialogues, especially via ICTs. Higher education institutions benefit significantly from the expanded use of ICTs technology in the classroom, including discussing current events. Scholars note both strengths and weaknesses of this move; for instance, Grosseck (2009) notes they allow increased access to, the creation of, and sharing of information, and cost savings to the University, but also pose challenges related to the technology (e.g., privacy settings) and the requirement for high-speed internet.

ICTs are not new to higher education. Venkatesh et al. (2013) point out that North America and European countries had already reached a "tipping point" (p. 6); the use of ICTs is going beyond individual instructors to having support at the institutional level. Venkatesh et al. (2013) continue that "…we are also witnessing a growing trend to incorporate increasingly sophisticated ICT tools in education. These may be signs of future indispensability …" (p. 8). As seen in our own use of ICTs in the classroom (distance or traditional), ICTs have been and will continue to be increasingly important to teaching in the professional disciplines. Additionally, research by Simándi (2018) has shown the use of ICTs technologies in adult education facilitates life-long learning, which "… builds on self-defined and self-regulating learning; the facilitator is present in the creation of the learning environment and supports the learning process as well" (p. 69). This development of life-long learning is a crucial component to professional disciplines, given that graduates require continuous knowledge updates throughout a career.

### COVID-19 and social change movements of 2020: a mandate for innovation

The current coronavirus pandemic (COVID‐19) has shifted secondary education to a predominantly online model. This shift is forecasted to impact secondary education for up to the next five-years (Dennis, 2020). Thus, educators must develop assessment tools that accurately evaluate learners' competency within digital spaces (Eltayar et al., 2020). Because online learning can increase a sense of isolation (Carolan et al., 2020), instructors need to create opportunities for students to build community and to grow their interpersonal communication skills when using technology. Peer-engagement in a virtual community of practice can mitigate learners' feelings of isolation associated with online learning (Carolan et al., 2020).

Beyond its impacts on secondary education, the COVID-19 pandemic has changed our collective daily lives. The pandemic has accelerated trends where people increasingly rely on technology to replace place-based activities, such as meeting with peers, running errands, and other daily living activities (Budd et al., 2020). COVID-19 has forced institutions and individuals to rely on new ways of communicating and connecting, often by harnessing ICTs platforms (Garfin, 2020).

Further, in the context of COVID-19, massive social unrest associated with ongoing police brutality and the Black Lives Matter movement has underscored the utility of ICTs social media platforms (Wilkins et al., 2019). Individuals and groups have used social media to quickly and easily organize, mobilize, and learn about world events, including protests and demonstrations. Technology and social media are now ubiquitous aspects of daily news in human life; educators must equip students with the skills to use technology and how it can be harnessed constructively for their personal and professional use.

Because instructors in higher education are now rethinking their content-delivery methods, activities and assignments need to fit in a world where pandemics might become part of a recurring landscape. Bao (2020) identified five high-impact principles for online education at the start of the COVID-19 pandemic in China, one of which was the creation of "high-quality participation to improve the breadth and depth of student's learning" (p. 1). Instructors can harness the dynamic and social aspects of ICTs to create high-impact activities and rigorous assignments to address a wide range of learning outcomes. Gurukkal (2020) states that COVID-19 will create a new "radical systemic transformation" (p.92) in higher education and that this transformation will have online teaching and evaluation at the forefront of educational delivery that makes-competency evaluation equal to other methods of assessment.

## Heutagogy

The study of adult learning has transformed over the years (Blaschke & Hase, 2019). Knowles’ (1973) theory of adult learning, referred to as andragogy, focuses on unique characteristics of adult learners including changes in self-concept, orientation, and readiness to learn from experience. Pedagogical learning is teacher-centric, and although andragogy focuses on the needs of the adult learner, it is still teacher-directed (Blaschke & Hase, 2019). Heutagogy is defined as "a form of self-determined learning…" (Blaschke, 2012), which focuses on developing students' "capability and capacity to learn" (Blaschke, 2012, p 2). Self-determined learning focuses on the process of learning (heutagogy) while self-directed learning focuses more on content (andragogy) (Blaschke & Hase, 2019). In summary, pedagogy, where the instructor is in control, can be viewed as one end of a continuum, with andragogy, where control is shared, in the middle of the continuum, and heutagogy can be viewed as the learner-determined end of the continuum (Blaschke & Hase, 2019). The heutagogical approach is the best fit with the learner-centered, unstructured student interaction that occurs when using ICTs, and offers practical boundaries to help educators think about how to apply these types of assignments in the classroom.

Prior research has explored the ways that emerging theories inform the use of ICTs from a heutagogical standpoint in the classroom. Blaschke and Hase (2019) identify these theories as complexity, connectivism, and rhizomatic (organic non-linear) theories, as students reflect on and construct their own knowledge through navigating non-linear tasks while connected and getting feedback with those outside their traditional classroom environments. Cochrane and Antonczak (2013) add humanism as a grounding theory when using this approach.

Although these are promising outcomes, ICTs may be perceived as a threat to traditional teacher-led knowledge transfer, and take new learning for teachers to reach the desired outcomes (Sutherland, 2004), including about how to prepare students for these types of assignments and provide an appropriate assessment.

It centers “—learner agency, self-efficacy, and capability, reflection and metacognition, and non-linear learning” (Blaschke & Hase, 2019, p. 1), and therefore its application lends well to college classrooms where critical thinking for lifelong learning is a highly-valued learner outcome. Further, heutagogy encourages competency and capability development. Competency refers to a learner’s ability to gain knowledge and skills, their confidence in their ability to solve problems, and how they apply acquired knowledge and skills in new and unfamiliar contexts (Blaschke, 2012). Competency-based education, with its focus on assessing students’ abilities to demonstrate predetermined knowledge, skills, and values, is common and often mandated in professional programs such as medicine, law, and social work (Frank et al., 2010; Morcke et al., 2013). While educators in professional programs may not always have flexibility in what is taught or how it is taught, a mindset grounded in heutagogy and the application of heutagogical principles whenever possible, offer students the opportunity to develop self-efficacy, creativity, and communication and collaboration skills (Blaschke, 2012).

For example, in the heutagogical classroom, much of the learning is student-driven rather than traditional instructor-led learning. The student takes a more active role in determining what is to be learned based on their own needs and interests (Glassner & Back, 2020), and the instructor acts as a facilitator. Students are able to dictate the learning process, which often results in non-linear learning where students not only reflect on outcomes but also on the learning process itself (Blaschke, 2012; Narayan et al., 2019). Heutagogy aims to develop lifelong learners, making it an excellent theoretical fit for the fields of applied sciences like nursing, social work, and public health, all disciplines that routinely change with the development of new knowledge, devices, techniques, and research into best practices (Bhoyrub et al., 2010). Specifically, heutagogical approaches foster the development of skills relevant to any professional setting, including communication skills, digital literacy, the ability to work with others to solve complex problems, metacognitive skills associated with a deeper self-understanding for how one learns, and self-confidence to apply skills in various settings (Blaschke, 2012). A heutagogical approach also emphasizes collaborative learning, particularly in which learners belong to communities of practice that exist as part of larger systems (Blaschke, 2012). These communities share new knowledge, experiences, and resources, which enhance all community members' capabilities. This form of ongoing learning requires engagement within and between members to develop knowledge that, over time, transforms learning and practice within larger systems (McDonald & Cater-Steel, 2016). This student-centered learning allows for meta-cognition that is transferable to other spaces, and when used in social media assignments, has been found to increase student familiarity with new tools and a sense of competency (Blaschke, 2014). Allowing learners to participate in communities of practice while in higher education allows for the development of relationships and social capital within these communities, which will support lifelong learning after formal education (Halsall et al., 2016). These communities of practice become personalized learning environments and must include tools that facilitate the learner's "three basic cognitive processes: reading, reflecting and sharing" (Torres Kompen et al., 2019, p. 196).

## Heutagogical principles for the use of ICTs assignments

ICTs assignments are unique in various ways, such as providing students access to larger online communities, encouraging creativity and self-directed learning, and being directed to public-facing audiences rather than solely the instructor. ICTs assignments can be created and evaluated using principles of heutagogy. McAuliffe et al. (2009) proposed the following four principles of heutagogy:

1. Understanding how to learn is crucial;
2. Educators should focus on the process instead of content;
3. Learning encompasses multiple disciplines; and
4. Learning should be self-chosen and self-directed.

Through ICTs assignments, students create products that conceivably have value beyond the classroom, which can influence student motivation and focus. By using ICT, students can be self-directed, develop or join communities of practice, and create content for these authentic audiences. Given these assignments' unique nature, viewing them through a traditional pedagogical or an andragogical framework is insufficient. Heutagogy emphasizes student-directed learning, a multidisciplinary point of view, and an examination of the process by which each student learns, which fits with the nature and scope of ICT assignments. Table 1 offers a visual of heutagogical principles for the use of ICTs assignments, which is described in the following paragraphs.

**Table 1:** Heutagogical Principles for the Use of ICTs Assignments

Principles of heutagogy	ICT applications	Authentic audience	Examples
Knowing how to learn	Students can learn how to use ICT tools through online videos, instruction guides, and tutorials. Instructors can use reflections to help the student understand their learning	Students can interact with content developers of tutorials or online help forums to ask questions, using screenshots to document their process for instructors	Students use online tutorials and guides to learn about voice recording software for a podcast assignment Students listen to podcasts and watch podcast creation videos to learn best practices in creating podcasts
Focus on process rather than content	ICT assignments often have multiple components that require students to utilize project management skills and focus on process	Students can beta-test and engage with their authentic audience early in the creation of their assignment, allowing for feedback at multiple points	A student researches prominent thought leaders on a given topic, interviews that thought leader, and then produces a video synthesizing the findings
Learning is multidisciplinary	ICT assignments can require students to create products designed to appeal to larger audiences, including other disciplines	Students can communicate with a variety of individuals outside of the classroom	Students create infographics designed to be shared on a social networking site and to communities of practice that compromise multiple disciplines
Learning is self-directed	ICT assignments can allow students to use self-chosen technology to produce content creatively	ICTs facilitate communication with individuals 24/7 and across geographical boundaries	For a final presentation, students are able to choose the format with the following choices: E-poster, podcast, infographic, or video

### Knowledge of the learning process

ICT assignments often require students to use a new digital and social technology to create some product or digital content; this enables them to learn at multiple levels. The students learn how to use the new technology, display or integrate their content ideas using the technology, and align them with their professional practice standards or learning objectives. For instance, when creating a podcast for an assignment, students must first learn the elements of a quality podcast, how to record and edit an audio file, and develop interview questions or outline a storyboard. They also consider how the content aligns with publicly representing themselves in relation to the course topic. This form of assignment offers a self-reflection opportunity about the process of learning, including an understanding of the audience, technology, sources of information that inform the work, and considerations for sharing it.

### Focus on process instead of content

ICT assignments often have multiple steps that lead to the creation of a piece of digital content. For example, Hitchcock et al., (2021) created an assignment where students interview local leaders and create a podcast. In this assignment, students need to learn how to use podcasting technology, search for local community leaders, learn how to write appropriate interview questions that match their disciplinary values and ethics, conduct an interview, edit the audio into a podcast and then reflect on the learning experience. By asking students to reflect on the assignment, instructors can motivate students to focus on their learning process and the skill development associated with the assignment's different parts.

### Multidisciplinary learning

ICT technology has made the world much more interconnected, allowing students to connect via social networking sites with experts or global colleagues within and outside of their discipline through digital tools that support communities of practice (Davis, 2015). Further, by requiring students to learn or become proficient in various forms of ICTs, they must think about their potential audiences' disciplinary perspectives while reflecting on their professional jargon, who they want to reach, and that person's experiences; Twitter, in particular, requires direct, clear, and concise communication (Davis, 2015). When students produce content designed for authentic audiences, they can create content that can appeal to a multidisciplinary audience.

### Self-directed learning

Knowles (1975) describes self-directed learning as "a process in which individuals take the initiative, with or without the help of others, in diagnosing their learning needs, formulating learnings goals, identifying human and material resources for learning, choosing and implementing appropriate learning strategies, and evaluating learning outcomes" (p. 18). ICT assignments can be structured in ways that promote self-directed learning, both in terms of content and process (Candy, 2004). Instructors can create assignments that allow students to choose the type of technology they use and their content. For example, an assignment focused on data collection and analysis could ask students to use any online platform to conduct a survey of their choosing and then analyze the results. In terms of content, students can approach their content development in a number of ways, perhaps choosing to produce their findings in a video, podcast, infographic, or traditional paper. Further, by understanding the principle of self-directed learning, educators can support students in professional education programs to develop and practice life-long learning skills by helping learners to identify specific topics, social problems, or populations in need of care that arouse their interest and curiosity (i.e. HIV/AIDS, domestic violence or working with children in foster care).

## Evaluation of ICTs assignments using a heutagogical approach

Educators in the professional disciplines must address ever-changing practice environments without the foresight of knowing what future needs may arise within a given professional field of practice. Competency-based education, with its focus on assessing students’ abilities to demonstrate predetermined knowledge, skills, and values, is common and often mandated in professional programs such as medicine, law, and social work (Frank et al., 2010; Morcke et al., 2013; Robbins, 2014). Historically, practice-based learning was used to prepare students for learning how to work within their profession. Still, it did little to prepare learners to adapt to systematic changes and the progression of theory and evidence-based practices. The traditional teaching model of subject expert and learner can only prepare learners for the currently-known dynamics within a profession (Bhoyrub et al., 2010). As previously noted, by integrating ICTs assignments from a heutagogical perspective, educators in professional programs can better prepare students for life-long learning throughout a career.

The value of using authentic audiences, such as proposed in this paper, is that they allow the learner to shift their focus from simply conveying their understanding of the subject matter to focusing on engaging their audience as it relates to their subject matter. This shift requires them to not only know their subject matter but what others in the audience already know and how to enhance their audience's knowledge related to the subject matter, while they might also learn from their authentic audience (Novakovich & Long, 2013). This shift increases the learners' engagement with the subject and simultaneously with others within their profession.

A conceptual approach for designing ICT assignments through a heutagogical lens should follow a logical path and allow the learner ownership over the design, content, and identification of an authentic audience to connect with and exchange information. The creation of an assessment tool for these assignments should include input from the learners as they conceptualize their ICT assignments' implementation. ICTs provide learners with multiple opportunities to develop new skill sets and develop expertise within their professional communities (Barber et al., 2015). ICT assignments expand the traditional use of problem-based learning commonly used in a conventional social learning framework by connecting the learner with collective knowledge beyond the traditional classroom and in environments where technology and expertise can outpace an instructor's ability to learn and integrate these advances.

### Evaluating ICT assignments designed with an heutagogical approach

The Framework for Authentic Intellectual Work (AIW) offers a starting point to think about how to assess ICT assignments (Newmann et al., 1996). Designed for use in K-12 education, this framework encourages the use of classroom assignments that model work completed by adults in their everyday work lives as a way to help students engage in genuine and rigorous learning. Multiple research from primary and secondary education in the US has documented the use of the AIW framework with both traditional and ICTs assignments (i.e. written paper vs. student-created videos), and overall, findings showed that AIW framework allowed educators to assess student learning outcomes across a variety of topics and disciplines (Newmann et al., 1996, 2001; Swan & Hofer, 2013). While not historically used in higher education, the framework supports the process of inquiry-based learning as well as creating works for public audiences, both common teaching strategies in higher education (Spires & Hervey, 2011). AIW includes three primary criteria for assessing an assignment. When all three criteria are met in an assignment's design, then the more authentic the student's work.

The first criterion of the AIW framework is the Construction of Knowledge, which could be compared to the lower-order thinking skills such as remembering and understanding key concepts (Anderson & Krathwohl, 2001). To meet this item, an assignment would require students to identify, interpret, and organize prior knowledge and new learning around a topic. For example, an instructor might ask students to create a podcast about how a social problem impacts their community (e.g., homelessness, food insecurity, etc.). To evaluate a student's podcast for knowledge construction, an instructor might look for how the student showed their understanding of the social problem (i.e., give facts about the problem using quality evidence) and how well they analyzed this social problem in their community (i.e., constructing arguments, considering alternative points of view and/or describing patterns). Additionally, students may need to learn technology skills with multimedia-based assignments such as recording and editing audio for a podcast, which aligns with the heutagogical principle of Knowledge of the Learning Process (see Table 1 for examples).

The second criterion is Disciplined Inquiry, which has two parts. First, it requires students to apply their knowledge of facts, theories, and skills to understand a problem or issue deeply. Here, students engage with some of Bloom's higher-order thinking skills to analyze and evaluate a problem or topic (Anderson & Krathwohl, 2001). In our podcast assignment example, an instructor would still be focused on how well a student integrates conflicting information about the causes of the social problem or draws logical conclusions from the facts in their podcast. Second is what Newman et al. (1995; 1996) referred to as elaborated communication or demonstration, whereby students express their findings through complex forms of communication such as writing or an oral presentation. Viewed from Bloom's Taxonomy, the instructor assesses how well a student created a multimedia-based assignment, such as how well the student expressed their ideas through such outcomes as writing, dialogues, or visual representations. From a heutagogical perspective, students are focused on the process of creating the assignment rather than the content itself (see Table 1 for examples). Considering the same podcast assignment, producing a high-quality podcast about a complex topic that includes show notes would demonstrate both elaborate written and oral communications.

The final criterion is Value beyond School, which reflects both a product that resembles real-life work and student perception that the work is meaningful. Value can be found in assignments that address a current problem of significance, incorporate students' lived experiences, and/or require students to share their work outside of the classroom. This also reflects the heutagogical principle that learning is multidisciplinary, moving beyond the classroom and curriculum (see Table 1 for examples). The previously discussed podcast assignment meets these criteria because it can be shared with individuals and groups outside of the classroom. Newmann et al. (2001) note that it is challenging to evaluate student performance if their work has value outside of the classroom because students nor educators have control over the potential audience, so this criterion only needs to be met in the assignment's design. However, social media analytics offer the potential to help students to assess the value of their work outside of the classroom. Analytics include metrics like the number of downloads of a file, the number of clicks on a hyperlink, or how many times a digital post or artifact has been re-shared within a social media platform (i.e., the number of retweets on Twitter). Additionally, an instructor can assess student satisfaction with the assignment as a tool that contributed to meaningful learning or request a reflection on how they engaged their public audience.

## Practical rubric for heutagogy-based assessment

Table 2. offers a sample rubric for a podcast assignment using the three AIW criteria, paired with specific assignment criteria and performance benchmarks adapted from the VALUES Rubrics created by the Association of American Colleges and Universities (AACU, 2010). The VALUES Rubrics are designed to be adjusted by instructors with terminology that best fits disciplines and courses. Besides being used by instructors to assess the product's quality, this rubric offers the opportunity for self and peer assessment by using benchmarks as a checklist for completed tasks.

**Table 2:** Sample rubric for a podcast assignment using AIW criteria

AIW criterion	Sample criteria	Sample performance benchmarks
Construction of Knowledge—Social Problem (Heutagogical Principle—Knowing how to learn)	Student demonstrated understanding of the social problem and how well they analyzed this problem in relation to their community (i.e., constructing arguments, considering alternative points of view, and/or describing patterns)	Clearly defines and comprehensively explains the social problem in the context of the discipline or class-related material Includes all relevant information necessary for full understanding by the target audience Evaluates the context of the social problem Incorporates multiple points of view into the podcast, such as expert knowledge and lived experience Conclusions are logical and reflect a synthesis of facts, context, and points of view Used credible information that is up to date (i.e., give facts about the problem using quality evidence) Provides appropriate attribution of information sources through show notes
Construction of Knowledge—Digital Literacy for Podcasting (Heutagogical Principle—Knowing how to learn)	Student demonstrated technology skills with multimedia-based assignments, such as how to record and edit audio for a podcast	Sound quality includes no background noise and distractions. Volume of voice, music, and effects enhance the presentation Length of podcast meets assignment requirements and keeps the listener interested and engaged (editing quality) The podcast contains no audio or content distractions Editing is used to improve quality, such as deleting "umms" or long pauses Sound effects enhance the podcast
In-Depth Understanding (Disciplinary Inquiry) (Heutagogical Principle—Focus on the Process rather than Content)	The student completed an interview with a content expert, service provider, public official, and/or person with a lived experience, incorporating the profession's ethical values	Questions are designed with an audience in mind, clear goals, and are open-ended Initial questions are thoughtful and draw out information from the person Questions demonstrate knowledge of the topic and draw upon literature and current events Questions use a disciplinary lens, linking to the profession, and offer considerations for professional values and ethics Follow-up questions promote clarification The interview is thoughtful and well-rehearsed, with smooth delivery of questions in a conversational style Highly effective enunciation, and the presenter's speech is clear and intelligible Expression and rhythm engage the listener
Elaborated Communication or Demonstration (Disciplinary Inquiry) (Heutagogical Principle—Focus on the Process rather than Content)	Student uses best practices to structure the podcast from start to end	Catchy and clever introduction that considers the interests of the target audience Provides relevant information and establishes a clear purpose that engages the listener immediately Identifies speaker, as well as date podcast, was produced and location of the speaker Keeps focus of podcast on the topic Conclusion clearly summarizes key information The language, professional jargon, slang, etc., considers the knowledge and preferences of the anticipated audience
Value Beyond School—Podcast Analytics (Heutagogical Principle—Learning is multidisciplinary)	Analytics show that a public audience is listening and rating the podcast	Number of downloads Number of listening starts Total time listened to the podcast Location and other demographics of listeners
Value Beyond School—Subjective Review (Heutagogical Principle—Learning is multidisciplinary)	Comments or reviews provided by a peer, content expert, or general public listener	Podcast ratings or reviews Testimonial from general public listener or review from content expert Number of social shares and recommendations

As can be seen, the rubric offers a heutagogical approach to assessment for instructors who want to gauge student learning with public-facing assignments. First, there are multiple assessment criteria in the rubric, from learning more about a social problem to integrating best practices for podcasting, encouraging students to focus on the process of creating a podcast from content to technical aspects. Second, because a podcast is designed for an audience outside the classroom, students need to consider that listeners will be diverse and incorporate multiple perspectives into their final product. Finally, the rubric is flexible enough to accommodate other forms of digital technology such as a video, should an instructor want to allow more technology options such as a video about a social problem or a vlog (a blog post that is shared as a video) (Tetloff et al., 2014). Blaschke (2014) offers additional considerations for a social media assignment based in self-determined learning, such as adjusting the assignment to the level of the student, incorporating self-reflection, and negotiating the assessment process. Student participants in Blaschke’s (2014) study informed other suggestions based on their perspectives, including providing guidance and support for new social media use, being prepared for those opposed to social media, making expectations clear, preparing how to track student activity, and assuring work is clearly aligned with learning objectives and the students’ future work.

## Implications for the higher education classroom

The integration of heutagogy with ICTs offers educators several opportunities to better prepare adult learners for future work environments in the twenty-first century, from learning ICT skills to developing the knowledge and attitudes needed for lifelong learning. For twenty-first century skills, numerous stakeholders from education and industry have weighed in based on expertise and experience (Battelle for Kids, 2020; Blaschke, 2014; Davidson, 2011; Trilling & Fadel, 2009). 21st-century skills now require digital literacy skills associated with ICTs assignments as well as career and learning self-reliance, collaboration skills, and creativity (Trilling & Fadel, 2009), all skills supported by the heutagogical design and assessment elements discussed in this paper.

Moreover, the heutagogical approach to learning and assessment helps instructors respond to the question, "why are you using social media in the classroom, and how do you assess it?" Here we clearly articulate why one would use social media, given students' need to engage in self-directed learning and knowledge generation to help prepare them for their future learning and technology-mediated engagement in a digital society. A gap in this work is how one assesses such varied activity; by dismantling the theory of heutagogy and authentic audiences, we land on a flexible but defendable approach that encourages intellectual freedom and autonomy while also holding students accountable for articulating the merits of their work.

Although the approach presented here offers insight into the ways to assess ICTs in the classroom through the lens of heutagogy, this article does not explore the degree of understanding that instructors or students need to have in ICTs in order to benefit from an ICT-mediated assignment, nor does it investigate technology access issues for students. Therefore, this approach for assessment may be ideally suited for instructors who are already using ICTs in the classroom and have thought through access concerns, but would benefit from structure to help assess and provide feedback to students about their learning. Additionally, this approach suggests flexibility and negotiation of learning outcomes, which likely has a time impact for instructors, an issue not evaluated in this article. Finally, the approach offered presents recommendations based on a review of the literature, and is not applied. Future research can assess the ways that students and teachers experience this approach, and whether it helps assess the desired learning outcomes.

## Data Availability

Not applicable.
